# Supplementary material for: A multivariate analysis of women's mating strategies and sexual selection on men's facial morphology
Source: R Soc Open Sci. 2020 Jan 15;7(1):191209. doi: 10.1098/rsos.191209 (PMC7029899; doi:10.1098/rsos.191209)
Supplement: Figure S1 and Table S1 [file rsos191209supp1.pdf]

A multivariate analysis of individual differences in women's mating strategies and sexual selection on men's facial masculinity and beardedness.

Tessa R. Clarkson[1]; Morgan J. Sidari[1]; Rosanna Sains[1]; Meredith Alexander[1]; Melissa Harrison[1]; Valeriya Mefodeva[1]; Samuel Pearson[1]; Anthony J. Lee[2]; Barnaby J. W. Dixson[1,3].

Electronic Supplementary Materials

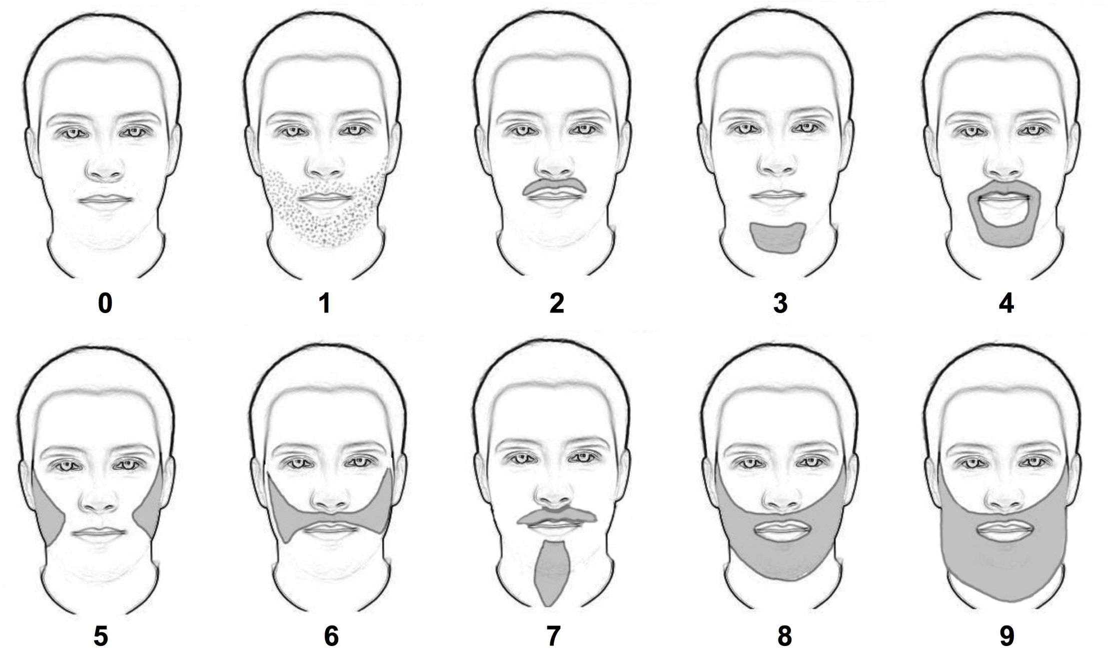

Figure S1. Each profile picture was scored as the most appropriate of ten possible facial hair styles: 0 = clean-shaven, 1 = stubble, 2 = moustache, 3 = goatee (without moustache), 4 = Goatee (with moustache), 5 = Sideburns, 6 = Sideburns and moustache, 7 = moustache and soul patch, 8 = Full beard (trimmed), 9 = Full beard (bushy)

Table S1. Analyses of reproductive ambition, current relationship status (Married/committed relationship vs single/dating) and women's preferences for facial masculinity and beardedness.

	Estimate (Std Error)	t-value (approx df.)	p-value
Intercept	37.95 (.89)	42.45 (14.14)	<.001***
Pregnancy Ambition	.95 (.72)	1.31 (651.32)	0.189
Facial Masculinity	1.46 (.13)	11.66 (915.12)	<.001***
Short/Long-Term	.96 (.36)	2.67 (914.89)	.008**
Relationship Status	-4.78 (1.50)	-3.19 (140.54)	.002**
Beardedness	5.47 (.54)	10.19 (913.78)	<.001***
Pregnancy Ambition * Facial Masculinity	-.09 (.13)	-.73 (915.12)	0.465
Pregnancy Ambition * Short/Long-Term	.61 (.36)	1.68 (914.89)	0.093
Facial Masculinity * Short/Long-Term	.61 (.14)	4.50 (11899.91)	<.001***
Pregnancy Ambition * Relationship Status	-1.64 (1.44)	-1.15 (875.17)	0.252
Facial Masculinity * Relationship Status	.08 (.25)	.31 (915.12)	0.753
Short/Long-Term * Relationship Status	-.61 (.72)	-.85 (914.89)	0.393
Pregnancy Ambition * Beardedness	-.82 (.54)	-1.52 (913.78)	0.129
Short/Long-Term * Beardedness	1.05 (.47)	2.25 (915.10)	.024*
Relationship Status * Beardedness	-.66 (1.07)	-.62 (913.78)	0.538
Pregnancy Ambition * Facial Masculinity * Short/Long-Term	.01 (.14)	.04 (11899.91)	0.97
Pregnancy Ambition * Facial Masculinity * Relationship Status	.20 (.25)	.78 (915.12)	0.437
Pregnancy Ambition * Short/Long-Term * Relationship Status	-1.74 (.72)	-2.41 (914.89)	.016*
Facial Masculinity * Short/Long-Term * Relationship Status	-.01 (.27)	-.04 (11899.91)	0.967
Pregnancy Ambition * Short/Long-Term * Beardedness	.62 (.47)	1.31 (915.10)	0.19
Pregnancy Ambition * Relationship Status * Beardedness	3.77 (1.08)	3.49 (913.78)	.001**
Short/Long-Term * Relationship Status * Beardedness	-.66 (.93)	-.70 (915.10)	0.482
Pregnancy Ambition * Facial Masculinity * Short/Long-Term * Relationship Status	.31 (.27)	1.12 (11899.91)	0.261
Pregnancy Ambition * Short/Long-Term * Relationship Status * Beardedness	-.15 (.94)	-.16 (915.10)	0.872
