# Supplementary material for: A multivariate analysis of women's mating strategies and sexual selection on men's facial morphology
Source: R Soc Open Sci. 2020 Jan 15;7(1):191209. doi: 10.1098/rsos.191209 (PMC7029899; doi:10.1098/rsos.191209)
Supplement: RMarkdown [file rsos191209supp2.pdf]

A multivariate analysis of women's mating strategies and sexual selection on masculinity in men's facial morphology.

Setup

Load Packages

```
library(tidyverse)
```

```
## -- Attaching packages --------------------------------------------------------------- tidyverse 1.2

## v ggplot2 3.1.0     v purrr   0.3.2
## v tibble  2.1.3     v dplyr   0.8.2
## v tidyr   0.8.2     v stringr 1.4.0
## v readr   1.3.1     v forcats 0.3.0

## -- Conflicts ------------------------------------------------------------------ tidyverse_conflicts
## x dplyr::filter() masks stats::filter()
## x dplyr::lag()    masks stats::lag()
```

```
library(lme4)
```

```
## Loading required package: Matrix

##
## Attaching package: 'Matrix'

## The following object is masked from 'package:tidyr':
##
##     expand
```

```
library(lmerTest)
```

```
##
## Attaching package: 'lmerTest'

## The following object is masked from 'package:lme4':
##
##     lmer

## The following object is masked from 'package:stats':
##
##     step
```

```
library(ICC)
```

Custom Functions

```
z <- function(x,remove.outliers = FALSE,winsorise = FALSE){
    out <- (x - mean(x,na.rm = TRUE))/sd(x,na.rm = TRUE)
    if (remove.outliers == TRUE){
```

```
      out <- ifelse(out >3,NA,ifelse(out < -3,NA,out))
    }
    if (winsorise == TRUE){
      out <- ifelse(out > 3,3,ifelse(out < -3,-3,out))
    }
    return(out)
}

correlations <- function(.data,variables,with=variables){
    .data <- as.data.frame(.data)
    vnames <- vector(mode="character",length=4*NROW(variables))
    for (v in 1:NROW(variables)){
        vnames[((v-1)*4)+2] <- variables[v]
    }
    output <- data.frame("variable"=vnames)
    for (w in 1:NROW(with)){
        print <- vector(mode="character",length=4*NROW(variables))
        for (v in 1:NROW(variables)){
            cor <- cor.test(.data[,variables[v]],.data[,with[w]])
            print[((v-1)*4)+2] <- paste("r = ",round(cor$estimate,3),sep="")
            print[((v-1)*4)+3] <- paste("p = ",round(cor$p.value,3),sep="")
            print[((v-1)*4)+4] <- paste("N = ",round(cor$parameter,3)+2,sep="")
        }
        output <- cbind(output,print)
    }
    colnames(output) <- c("variables",with)
    output
}

descriptives <- function(data,variables){
    output <- data.frame("variable"=variables,"N"=NA,"min"=NA,"max"=NA,"mean"=NA,"sd"=NA)
    d <- as.data.frame(data[variables])
    for (v in 1:NROW(variables)){
        output[v,"N"] <- sum(!is.na(d[v]))
        output[v,"min"] <- min(d[,v],na.rm=TRUE)
        output[v,"max"] <- max(d[,v],na.rm=TRUE)
        output[v,"mean"] <- mean(d[,v],na.rm=TRUE)
        output[v,"sd"] <- sd(d[,v],na.rm=TRUE)
    }
    print(output)
    return(data)
}

recode_masculinity <- function(x){
    out <- recode(x,    "40" = "-1.2649111",
                        "70" = "-0.6324555",
                        "100" = "0.0000000",
                        "130" = "0.6324555",
                        "160" = "1.2649111") %>%
        as.numeric()
    return(out)
}
```

Load Data

```
data <- read.csv('data_tessa.csv',stringsAsFactors = FALSE) %>%
    mutate(ID = row_number())
data.stimuli <- read.csv('coding_tessa.csv',stringsAsFactors = FALSE)
```

Data Setup

Participant-Level Data

```
data.participant <- dplyr::select(data,ID,sex = MaleorFemale,age = Q23_1,rs = Q6,fathers_beards:Pregancy
  descriptives(c("age","Tybur_Pathogen","Tybur_Sexual","Tybur_Moral","Heebie_Jeebies","Mate_Value","Preg
    mutate( Tybur_Pathogen = z(Tybur_Pathogen),
            Tybur_Sexual = z(Tybur_Sexual),
            Tybur_Moral = z(Tybur_Moral),
            Heebie_Jeebies = z(Heebie_Jeebies),
            Mate_Value = z(Mate_Value),
            Pregancy_Ambition_item_removed = z(Pregancy_Ambition_item_removed),
            fathers_beards = z(fathers_beards),
            partners_beards = z(partners_beards),
            age = z(age),
            rs = recode(rs,"Dating" = -.5,
                            "Married / Committed Relationship" = .5,
                            "Single" = -.5,
                            "Other" = -.5))
```

```
##                         variable   N   min   max      mean        sd
## 1                            age 919 18.00 70.00 37.190424 11.663196
## 2                 Tybur_Pathogen 919  0.14  6.00  4.133090  1.060478
## 3                   Tybur_Sexual 919  0.14  6.00  3.165756  1.368337
## 4                    Tybur_Moral 919  0.00  6.00  3.845952  1.531803
## 5                 Heebie_Jeebies 919  1.00  7.00  5.217965  1.486953
## 6                     Mate_Value 919  1.00  7.00  4.742383  1.173392
## 7 Pregancy_Ambition_item_removed 919  0.83  5.83  3.015462  1.417399
```

Correlations between participant level data

```
correlations(data.participant,c("Tybur_Pathogen","Tybur_Sexual","Tybur_Moral","Heebie_Jeebies"))
```

```
##          variables Tybur_Pathogen Tybur_Sexual Tybur_Moral Heebie_Jeebies
## 1
## 2  Tybur_Pathogen          r = 1    r = 0.459   r = 0.325      r = 0.465
## 3                          p = 0        p = 0       p = 0          p = 0
## 4                        N = 919      N = 919     N = 919        N = 919
## 5
## 6    Tybur_Sexual      r = 0.459        r = 1   r = 0.363      r = 0.246
## 7                          p = 0        p = 0       p = 0          p = 0
## 8                        N = 919      N = 919     N = 919        N = 919
## 9
## 10    Tybur_Moral      r = 0.325    r = 0.363       r = 1      r = 0.167
```

```
## 11                         p = 0        p = 0       p = 0          p = 0
## 12                       N = 919      N = 919     N = 919        N = 919
## 13
## 14 Heebie_Jeebies      r = 0.465    r = 0.246   r = 0.167          r = 1
## 15                         p = 0        p = 0       p = 0          p = 0
## 16                       N = 919      N = 919     N = 919        N = 919
```

```
correlations(data.participant,c("fathers_beards","partners_beards"))
```

```
##         variables fathers_beards partners_beards
## 1
## 2  fathers_beards          r = 1        r = 0.07
## 3                          p = 0       p = 0.101
## 4                        N = 754         N = 550
## 5
## 6 partners_beards       r = 0.07           r = 1
## 7                      p = 0.101           p = 0
## 8                        N = 550         N = 662
```

```
correlations(data.participant,c("age","Pregancy_Ambition_item_removed"))
```

```
##                          variables        age Pregancy_Ambition_item_removed
## 1
## 2                              age      r = 1                     r = -0.358
## 3                                       p = 0                          p = 0
## 4                                     N = 919                        N = 919
## 5
## 6 Pregancy_Ambition_item_removed r = -0.358                          r = 1
## 7                                       p = 0                          p = 0
## 8                                     N = 919                        N = 919
```

Rating-Level Data

```
data.ratings <- dplyr::select(data,ID,bavg17_s160_1:cavg11_s40_2) %>%
    gather(key = "stimuli_id",value = "rating",bavg17_s160_1:cavg11_s40_2) %>%
    separate(stimuli_id,into = c("stimuli","masc","sl_term"),sep = "_") %>%
    mutate( bearded = ifelse(substr(stimuli,1,1) == "b",.5,-.5),
            masc = recode_masculinity(as.numeric(substr(masc,2,4))),
            stimuli = as.numeric(substr(stimuli,5,6)),
            sl_term = ifelse(sl_term == 1,-.5,.5)) %>%
    arrange(ID,stimuli,masc,sl_term)
```

Join Datasets

```
data.analysis <- left_join(data.ratings,data.participant,by = "ID")
```

Run Linear Effect Models

Preference for masculinity and beards associated with age.

```r
model.0 <- lmer(rating ~ masc*age + bearded*age + (1 + masc + bearded|ID) + (1 + age| stimuli),data = da
```

```
## singular fit
```

```r
summary(model.0)
```

```
## Linear mixed model fit by REML. t-tests use Satterthwaite's method [
## lmerModLmerTest]
## Formula:
## rating ~ masc * age + bearded * age + (1 + masc + bearded | ID) +
##     (1 + age | stimuli)
##    Data: data.analysis
##
## REML criterion at convergence: 460715.9
##
## Scaled residuals:
##     Min      1Q  Median      3Q     Max
## -4.7337 -0.5049 -0.0548  0.4539  5.6472
##
## Random effects:
##  Groups   Name        Variance  Std.Dev. Corr
##  ID       (Intercept) 4.145e+02 20.35828
##           masc        8.532e+00  2.92100 -0.01
##           bearded     2.299e+02 15.16393  0.05  0.12
##  stimuli  (Intercept) 1.041e+00  1.02005
##           age         7.413e-05  0.00861 -1.00
##  Residual             2.156e+02 14.68289
## Number of obs: 55140, groups:  ID, 919; stimuli, 3
##
## Fixed effects:
##             Estimate Std. Error       df t value Pr(>|t|)
## (Intercept)  37.2594     0.8954  10.4119  41.612 6.43e-13 ***
## masc          1.4847     0.1190 916.9990  12.472  < 2e-16 ***
## age           3.3837     0.6748 916.7066   5.014 6.40e-07 ***
## bearded       5.6590     0.5156 917.0020  10.975  < 2e-16 ***
## masc:age      0.4148     0.1191 916.9990   3.483  0.00052 ***
## age:bearded  -0.9500     0.5159 917.0020  -1.841  0.06587 .
## ---
## Signif. codes:  0 '***' 0.001 '**' 0.01 '*' 0.05 '.' 0.1 ' ' 1
##
## Correlation of Fixed Effects:
##             (Intr) masc   age    beardd masc:g
## masc        -0.005
## age         -0.005  0.000
## bearded      0.036  0.093  0.000
## masc:age     0.000  0.000 -0.006  0.000
## age:bearded  0.000  0.000  0.047  0.000  0.093
## convergence code: 0
## singular fit
```

```r
data.analysis %>%
  mutate(cat.age = ifelse(age < 0,"Younger than mean","Older than mean")) %>%
  ggplot(aes(x = masc,y = rating,colour = cat.age)) +
  geom_smooth(method = "lm")
```

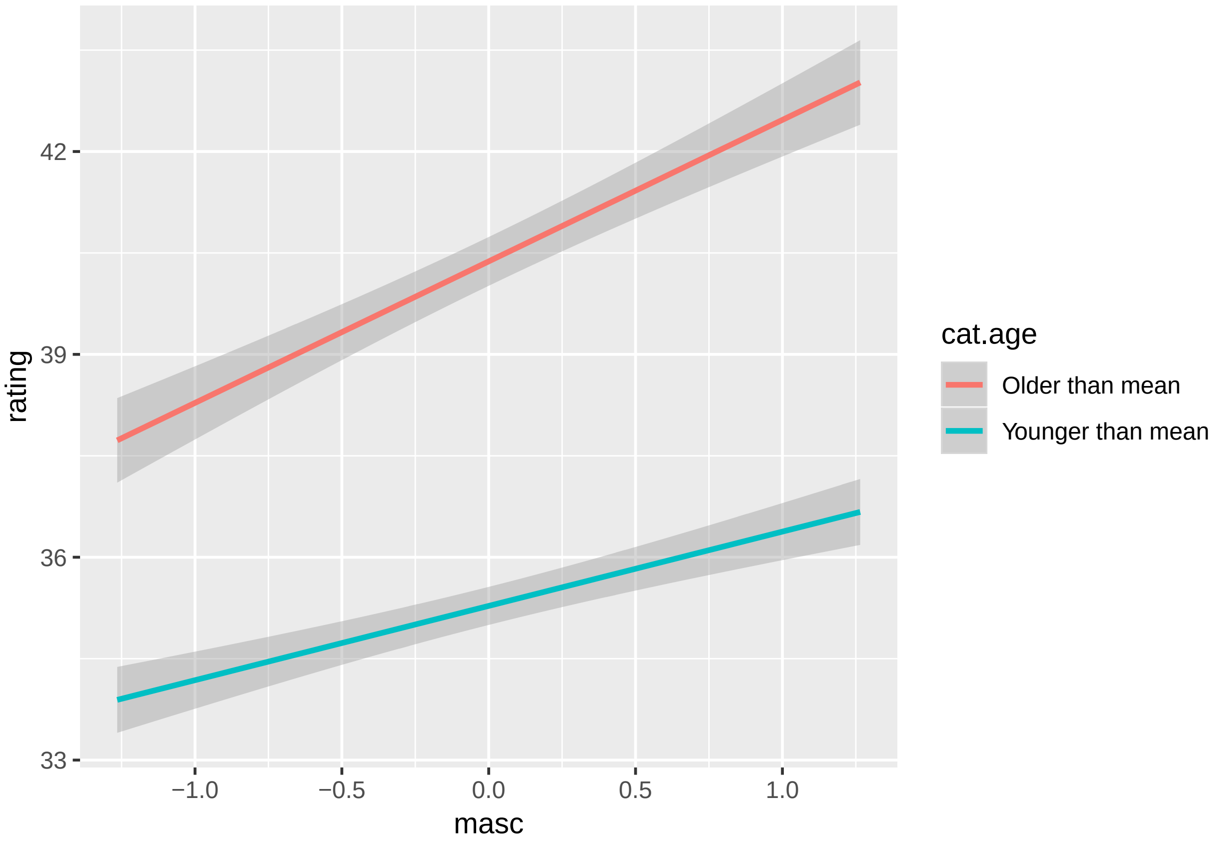

Disgust and Ectoparasite Avoidance on Preferences

```r
model1 <- lmer(rating ~     Tybur_Pathogen*masc*sl_term + Tybur_Pathogen*bearded*sl_term +
                        Tybur_Sexual*masc*sl_term + Tybur_Sexual*bearded*sl_term +
                        Tybur_Moral*masc*sl_term + Tybur_Moral*bearded*sl_term +
                        Heebie_Jeebies*masc*sl_term + Heebie_Jeebies*bearded*sl_term +
                        (1 + masc*sl_term + bearded*sl_term | ID) +
                        (1 + Tybur_Pathogen + Tybur_Sexual + Tybur_Moral + Heebie_Jeebies | stimuli),
            data = data.analysis)

save(model1,file = "model1.Rdata")
```

```r
summary(model1)
```

```
## Linear mixed model fit by REML. t-tests use Satterthwaite's method [
## lmerModLmerTest]
## Formula:
## rating ~ Tybur_Pathogen * masc * sl_term + Tybur_Pathogen * bearded *
##     sl_term + Tybur_Sexual * masc * sl_term + Tybur_Sexual *
##     bearded * sl_term + Tybur_Moral * masc * sl_term + Tybur_Moral *
##     bearded * sl_term + Heebie_Jeebies * masc * sl_term + Heebie_Jeebies *
##     bearded * sl_term + (1 + masc * sl_term + bearded * sl_term |
##     ID) + (1 + Tybur_Pathogen + Tybur_Sexual + Tybur_Moral +
```

```
##     Heebie_Jeebies | stimuli)
##    Data: data.analysis
##
## REML criterion at convergence: 454581.3
##
## Scaled residuals:
##     Min      1Q  Median      3Q     Max
## -5.8793 -0.4792 -0.0458  0.4242  6.1995
##
## Random effects:
##  Groups   Name            Variance Std.Dev. Corr
##  ID       (Intercept)     421.5809 20.5324
##           masc              9.3888  3.0641   0.01
##           sl_term          96.0298  9.7995   0.08 -0.05
##           bearded         217.1668 14.7366   0.03  0.09  0.02
##           masc:sl_term      0.4136  0.6432   0.40  0.63  0.50  0.02
##           sl_term:bearded 134.7791 11.6094   0.05  0.04  0.15  0.23  0.52
##  stimuli  (Intercept)       1.0566  1.0279
##           Tybur_Pathogen    0.4478  0.6692   1.00
##           Tybur_Sexual      0.1179  0.3434  -0.98 -0.98
##           Tybur_Moral       0.1001  0.3163   0.94  0.94 -0.86
##           Heebie_Jeebies    0.1303  0.3610  -0.96 -0.96  1.00 -0.81
##  Residual                 180.5670 13.4375
## Number of obs: 55140, groups:  ID, 919; stimuli, 3
##
## Fixed effects:
##                                  Estimate Std. Error         df t value
## (Intercept)                     3.726e+01  9.023e-01  1.029e+01  41.292
## Tybur_Pathogen                 -1.554e+00  9.349e-01  6.019e+01  -1.662
## masc                            1.485e+00  1.196e-01  9.143e+02  12.412
## sl_term                         7.599e-01  3.429e-01  9.140e+02   2.216
## bearded                         5.659e+00  4.994e-01  9.143e+02  11.331
## Tybur_Sexual                    2.537e-01  8.164e-01  3.187e+02   0.311
## Tybur_Moral                     2.489e+00  7.657e-01  3.343e+02   3.251
## Heebie_Jeebies                  3.125e-01  7.966e-01  2.633e+02   0.392
## Tybur_Pathogen:masc             8.193e-02  1.498e-01  9.143e+02   0.547
## Tybur_Pathogen:sl_term         -5.427e-01  4.295e-01  9.140e+02  -1.264
## masc:sl_term                    6.306e-01  1.297e-01  1.204e+04   4.861
## Tybur_Pathogen:bearded          1.275e+00  6.255e-01  9.143e+02   2.039
## sl_term:bearded                 9.609e-01  4.462e-01  9.148e+02   2.154
## masc:Tybur_Sexual              -1.880e-01  1.394e-01  9.143e+02  -1.349
## sl_term:Tybur_Sexual            1.341e+00  3.996e-01  9.140e+02   3.356
## bearded:Tybur_Sexual           -4.311e+00  5.819e-01  9.143e+02  -7.409
## masc:Tybur_Moral                3.116e-01  1.309e-01  9.143e+02   2.381
## sl_term:Tybur_Moral            -4.632e-02  3.752e-01  9.140e+02  -0.123
## bearded:Tybur_Moral             2.046e+00  5.464e-01  9.143e+02   3.745
## masc:Heebie_Jeebies            -4.018e-02  1.353e-01  9.143e+02  -0.297
## sl_term:Heebie_Jeebies         -1.750e-01  3.879e-01  9.140e+02  -0.451
## bearded:Heebie_Jeebies         -1.309e+00  5.649e-01  9.143e+02  -2.318
## Tybur_Pathogen:masc:sl_term    -1.654e-01  1.625e-01  1.204e+04  -1.018
## Tybur_Pathogen:sl_term:bearded  4.901e-01  5.588e-01  9.148e+02   0.877
## masc:sl_term:Tybur_Sexual      -2.453e-02  1.511e-01  1.204e+04  -0.162
## sl_term:bearded:Tybur_Sexual   -3.242e-01  5.198e-01  9.148e+02  -0.624
## masc:sl_term:Tybur_Moral        5.964e-02  1.419e-01  1.204e+04   0.420
```

```
## sl_term:bearded:Tybur_Moral    -4.953e-03  4.881e-01  9.148e+02  -0.010
## masc:sl_term:Heebie_Jeebies     1.958e-02  1.467e-01  1.204e+04   0.133
## sl_term:bearded:Heebie_Jeebies -5.992e-01  5.047e-01  9.148e+02  -1.187
##                                Pr(>|t|)
## (Intercept)                    9.06e-13 ***
## Tybur_Pathogen                 0.101781
## masc                            < 2e-16 ***
## sl_term                        0.026942 *
## bearded                         < 2e-16 ***
## Tybur_Sexual                   0.756170
## Tybur_Moral                    0.001268 **
## Heebie_Jeebies                 0.695191
## Tybur_Pathogen:masc            0.584619
## Tybur_Pathogen:sl_term         0.206708
## masc:sl_term                   1.18e-06 ***
## Tybur_Pathogen:bearded         0.041762 *
## sl_term:bearded                0.031521 *
## masc:Tybur_Sexual              0.177646
## sl_term:Tybur_Sexual           0.000823 ***
## bearded:Tybur_Sexual           2.89e-13 ***
## masc:Tybur_Moral               0.017482 *
## sl_term:Tybur_Moral            0.901764
## bearded:Tybur_Moral            0.000192 ***
## masc:Heebie_Jeebies            0.766584
## sl_term:Heebie_Jeebies         0.651907
## bearded:Heebie_Jeebies         0.020691 *
## Tybur_Pathogen:masc:sl_term    0.308555
## Tybur_Pathogen:sl_term:bearded 0.380665
## masc:sl_term:Tybur_Sexual      0.871089
## sl_term:bearded:Tybur_Sexual   0.533002
## masc:sl_term:Tybur_Moral       0.674316
## sl_term:bearded:Tybur_Moral    0.991906
## masc:sl_term:Heebie_Jeebies    0.893843
## sl_term:bearded:Heebie_Jeebies 0.235395
## ---
## Signif. codes:  0 '***' 0.001 '**' 0.01 '*' 0.05 '.' 0.1 ' ' 1
##
## Correlation matrix not shown by default, as p = 30 > 12.
## Use print(x, correlation=TRUE)  or
##     vcov(x)        if you need it
## convergence code: 1
## singular fit
## maxfun < 10 * length(par)^2 is not recommended.
```

Mate Value on Preferences

```
#Model with correlated random effects failed to converge.

model2b <- lmer(rating ~    Mate_Value*masc*sl_term + Mate_Value*bearded*sl_term +
                       (1 + masc*sl_term + bearded*sl_term || ID) +
                       (1 + Mate_Value || stimuli),
            data = data.analysis)
```

```r
save(model2b,file = "model2b.Rdata")
```

```r
summary(model2b)
```

```
## Linear mixed model fit by REML. t-tests use Satterthwaite's method [
## lmerModLmerTest]
## Formula: rating ~ Mate_Value * masc * sl_term + Mate_Value * bearded *
##     sl_term + (1 + masc * sl_term + bearded * sl_term || ID) +
##     (1 + Mate_Value || stimuli)
##    Data: data.analysis
##
## REML criterion at convergence: 454827.5
##
## Scaled residuals:
##     Min      1Q  Median      3Q     Max
## -5.8912 -0.4803 -0.0445  0.4243  6.1488
##
## Random effects:
##  Groups    Name            Variance  Std.Dev.
##  ID        (Intercept)     4.264e+02 2.065e+01
##  ID.1      masc            9.422e+00 3.070e+00
##  ID.2      sl_term         9.665e+01 9.831e+00
##  ID.3      bearded         2.326e+02 1.525e+01
##  ID.4      masc:sl_term    4.732e-12 2.175e-06
##  ID.5      sl_term:bearded 1.338e+02 1.157e+01
##  stimuli   (Intercept)     1.042e+00 1.021e+00
##  stimuli.1 Mate_Value      9.330e-02 3.054e-01
##  Residual                  1.809e+02 1.345e+01
## Number of obs: 55140, groups:  ID, 919; stimuli, 3
##
## Fixed effects:
##                              Estimate Std. Error         df t value
## (Intercept)                 3.726e+01  9.026e-01  1.075e+01  41.280
## Mate_Value                 -3.442e-01  7.063e-01  2.999e+02  -0.487
## masc                        1.485e+00  1.198e-01  9.170e+02  12.392
## sl_term                     7.599e-01  3.439e-01  9.170e+02   2.209
## bearded                     5.659e+00  5.159e-01  9.170e+02  10.968
## Mate_Value:masc            -6.821e-02  1.199e-01  9.170e+02  -0.569
## Mate_Value:sl_term          6.336e-01  3.441e-01  9.170e+02   1.841
## masc:sl_term                6.306e-01  1.281e-01  5.054e+04   4.923
## Mate_Value:bearded         -7.675e-01  5.162e-01  9.170e+02  -1.487
## sl_term:bearded             9.609e-01  4.451e-01  9.170e+02   2.159
## Mate_Value:masc:sl_term    -5.585e-02  1.282e-01  5.054e+04  -0.436
## Mate_Value:sl_term:bearded -8.292e-01  4.453e-01  9.170e+02  -1.862
##                            Pr(>|t|)
## (Intercept)                3.45e-13 ***
## Mate_Value                   0.6264
## masc                        < 2e-16 ***
## sl_term                      0.0274 *
## bearded                     < 2e-16 ***
## Mate_Value:masc              0.5695
## Mate_Value:sl_term           0.0659 .
## masc:sl_term               8.55e-07 ***
```

```
## Mate_Value:bearded           0.1374
## sl_term:bearded              0.0311 *
## Mate_Value:masc:sl_term      0.6630
## Mate_Value:sl_term:bearded   0.0629 .
## ---
## Signif. codes:  0 '***' 0.001 '**' 0.01 '*' 0.05 '.' 0.1 ' ' 1
##
## Correlation of Fixed Effects:
##              (Intr) Mat_Vl masc  sl_trm beardd Mt_Vl:m Mt_V:_ msc:s_
## Mate_Value  0.000
## masc        0.000  0.000
## sl_term     0.000  0.000  0.000
## bearded     0.000  0.000  0.000 0.000
## Mate_Vl:msc 0.000  0.000  0.000 0.000  0.000
## Mt_Vl:sl_tr 0.000  0.000  0.000 0.000  0.000  0.000
## masc:sl_trm 0.000  0.000  0.000 0.000  0.000  0.000   0.000
## Mat_Vl:brdd 0.000  0.000  0.000 0.000  0.000  0.000   0.000  0.000
## sl_trm:brdd 0.000  0.000  0.000 0.000  0.000  0.000   0.000  0.000
## Mt_Vl:msc:_ 0.000  0.000  0.000 0.000  0.000  0.000   0.000  0.000
## Mt_Vl:sl_t: 0.000  0.000  0.000 0.000  0.000  0.000   0.000  0.000
##             Mt_Vl:b sl_tr: M_V::_
## Mate_Value
## masc
## sl_term
## bearded
## Mate_Vl:msc
## Mt_Vl:sl_tr
## masc:sl_trm
## Mat_Vl:brdd
## sl_trm:brdd 0.000
## Mt_Vl:msc:_ 0.000   0.000
## Mt_Vl:sl_t: 0.000   0.000  0.000
## convergence code: 0
## singular fit
```

Pregnancy Ambition on Preferences

```
model3 <- lmer(rating ~     Pregancy_Ambition_item_removed*masc*sl_term*rs + Pregancy_Ambition_item_remo

                        (1 + masc*sl_term + bearded*sl_term | ID) +
                        (1 + Pregancy_Ambition_item_removed*rs | stimuli),
            data = data.analysis)

save(model3,file = "model3.Rdata")
```

```
summary(model3)
```

```
## Linear mixed model fit by REML. t-tests use Satterthwaite's method [
## lmerModLmerTest]
## Formula: rating ~ Pregancy_Ambition_item_removed * masc * sl_term * rs +
##     Pregancy_Ambition_item_removed * bearded * sl_term * rs +
##     (1 + masc * sl_term + bearded * sl_term | ID) + (1 + Pregancy_Ambition_item_removed *
##     rs | stimuli)
```

```
##    Data: data.analysis
##
## REML criterion at convergence: 454670.9
##
## Scaled residuals:
##     Min      1Q  Median      3Q     Max
## -5.8630 -0.4796 -0.0455  0.4252  6.1648
##
## Random effects:
##  Groups   Name                               Variance  Std.Dev. Corr
##  ID       (Intercept)                        421.72696 20.5360
##           masc                                 9.45663  3.0752   0.02
##           sl_term                             96.46947  9.8219   0.08 -0.05
##           bearded                            230.35130 15.1773   0.04  0.10
##           masc:sl_term                         0.41792  0.6465   0.42  0.62
##           sl_term:bearded                    134.62686 11.6029   0.04  0.04
##  stimuli  (Intercept)                          0.88414  0.9403
##           Pregancy_Ambition_item_removed       0.03979  0.1995   1.00
##           rs                                   0.70954  0.8423   0.89  0.89
##           Pregancy_Ambition_item_removed:rs    0.03894  0.1973  -0.40 -0.40
##  Residual                                    180.72131 13.4433
##
##
##
##
##   0.00
##   0.51  0.02
##   0.14  0.24  0.49
##
##
##
##  -0.78
##
## Number of obs: 55140, groups:  ID, 919; stimuli, 3
##
## Fixed effects:
##                                                  Estimate Std. Error
## (Intercept)                                     3.795e+01  8.939e-01
## Pregancy_Ambition_item_removed                  9.530e-01  7.247e-01
## masc                                            1.461e+00  1.253e-01
## sl_term                                         9.589e-01  3.590e-01
## rs                                             -4.782e+00  1.501e+00
## bearded                                         5.465e+00  5.365e-01
## Pregancy_Ambition_item_removed:masc            -9.234e-02  1.262e-01
## Pregancy_Ambition_item_removed:sl_term          6.087e-01  3.617e-01
## masc:sl_term                                    6.097e-01  1.356e-01
## Pregancy_Ambition_item_removed:rs              -1.644e+00  1.436e+00
## masc:rs                                         7.875e-02  2.506e-01
## sl_term:rs                                     -6.135e-01  7.179e-01
## Pregancy_Ambition_item_removed:bearded         -8.220e-01  5.405e-01
## sl_term:bearded                                 1.050e+00  4.659e-01
## rs:bearded                                     -6.615e-01  1.073e+00
## Pregancy_Ambition_item_removed:masc:sl_term     5.151e-03  1.366e-01
## Pregancy_Ambition_item_removed:masc:rs          1.962e-01  2.525e-01
```

```
## Pregancy_Ambition_item_removed:sl_term:rs         -1.741e+00  7.233e-01
## masc:sl_term:rs                                    -1.130e-02  2.711e-01
## Pregancy_Ambition_item_removed:sl_term:bearded      6.159e-01  4.694e-01
## Pregancy_Ambition_item_removed:rs:bearded           3.768e+00  1.081e+00
## sl_term:rs:bearded                                 -6.557e-01  9.318e-01
## Pregancy_Ambition_item_removed:masc:sl_term:rs      3.069e-01  2.732e-01
## Pregancy_Ambition_item_removed:sl_term:rs:bearded -1.509e-01  9.388e-01
##                                                           df t value
## (Intercept)                                         1.414e+01  42.453
## Pregancy_Ambition_item_removed                      6.513e+02   1.315
## masc                                                9.151e+02  11.662
## sl_term                                             9.149e+02   2.671
## rs                                                  1.405e+02  -3.185
## bearded                                             9.138e+02  10.187
## Pregancy_Ambition_item_removed:masc                 9.151e+02  -0.731
## Pregancy_Ambition_item_removed:sl_term              9.149e+02   1.683
## masc:sl_term                                        1.190e+04   4.498
## Pregancy_Ambition_item_removed:rs                   8.752e+02  -1.145
## masc:rs                                             9.151e+02   0.314
## sl_term:rs                                          9.149e+02  -0.855
## Pregancy_Ambition_item_removed:bearded              9.138e+02  -1.521
## sl_term:bearded                                     9.151e+02   2.254
## rs:bearded                                          9.138e+02  -0.617
## Pregancy_Ambition_item_removed:masc:sl_term         1.190e+04   0.038
## Pregancy_Ambition_item_removed:masc:rs              9.151e+02   0.777
## Pregancy_Ambition_item_removed:sl_term:rs           9.149e+02  -2.407
## masc:sl_term:rs                                     1.190e+04  -0.042
## Pregancy_Ambition_item_removed:sl_term:bearded      9.151e+02   1.312
## Pregancy_Ambition_item_removed:rs:bearded           9.138e+02   3.486
## sl_term:rs:bearded                                  9.151e+02  -0.704
## Pregancy_Ambition_item_removed:masc:sl_term:rs      1.190e+04   1.123
## Pregancy_Ambition_item_removed:sl_term:rs:bearded   9.151e+02  -0.161
##                                                    Pr(>|t|)
## (Intercept)                                        2.57e-16 ***
## Pregancy_Ambition_item_removed                     0.188989
## masc                                                < 2e-16 ***
## sl_term                                            0.007686 **
## rs                                                 0.001781 **
## bearded                                             < 2e-16 ***
## Pregancy_Ambition_item_removed:masc                0.464708
## Pregancy_Ambition_item_removed:sl_term             0.092710 .
## masc:sl_term                                       6.94e-06 ***
## Pregancy_Ambition_item_removed:rs                  0.252336
## masc:rs                                            0.753399
## sl_term:rs                                         0.392978
## Pregancy_Ambition_item_removed:bearded             0.128681
## sl_term:bearded                                    0.024461 *
## rs:bearded                                         0.537712
## Pregancy_Ambition_item_removed:masc:sl_term        0.969919
## Pregancy_Ambition_item_removed:masc:rs             0.437259
## Pregancy_Ambition_item_removed:sl_term:rs          0.016263 *
## masc:sl_term:rs                                    0.966767
## Pregancy_Ambition_item_removed:sl_term:bearded     0.189835
## Pregancy_Ambition_item_removed:rs:bearded          0.000514 ***
```

```
## sl_term:rs:bearded                                 0.481831
## Pregancy_Ambition_item_removed:masc:sl_term:rs    0.261266
## Pregancy_Ambition_item_removed:sl_term:rs:bearded 0.872376
## ---
## Signif. codes:  0 '***' 0.001 '**' 0.01 '*' 0.05 '.' 0.1 ' ' 1
```

```
##
## Correlation matrix not shown by default, as p = 24 > 12.
## Use print(x, correlation=TRUE)  or
##     vcov(x)        if you need it
```

```
## convergence code: 1
## singular fit
```

```r
model3a <- lmer(rating ~    Pregancy_Ambition_item_removed*masc*age + Pregancy_Ambition_item_removed*bea

                        (1 + masc + bearded | ID) +
                        (1 + Pregancy_Ambition_item_removed*age | stimuli),
            data = data.analysis)

save(model3a,file = "model3a.Rdata")
```

```r
summary(model3a)
```

```
## Linear mixed model fit by REML. t-tests use Satterthwaite's method [
## lmerModLmerTest]
## Formula:
## rating ~ Pregancy_Ambition_item_removed * masc * age + Pregancy_Ambition_item_removed *
##     bearded * age + (1 + masc + bearded | ID) + (1 + Pregancy_Ambition_item_removed *
##     age | stimuli)
##    Data: data.analysis
##
## REML criterion at convergence: 460681.4
##
## Scaled residuals:
##     Min      1Q  Median      3Q     Max
## -4.7263 -0.5062 -0.0532  0.4555  5.6555
##
## Random effects:
##  Groups   Name                               Variance  Std.Dev. Corr
##  ID       (Intercept)                        410.82875 20.2689
##           masc                                 8.51895  2.9187  -0.01
##           bearded                            229.86373 15.1613   0.05
##  stimuli  (Intercept)                          1.17942  1.0860
##           Pregancy_Ambition_item_removed       0.08591  0.2931  1.00
##           age                                  0.02119  0.1456  1.00
##           Pregancy_Ambition_item_removed:age   0.07633  0.2763  0.69
##  Residual                                    215.49847 14.6799
##
##
##
##   0.12
##
##
##  1.00
##  0.69  0.76
```

```
##
## Number of obs: 55140, groups:  ID, 919; stimuli, 3
##
## Fixed effects:
##                                             Estimate Std. Error        df
## (Intercept)                                 36.74518    0.95880  10.64652
## Pregancy_Ambition_item_removed               1.68957    0.74349 363.60706
## masc                                         1.40877    0.12852 915.00098
## age                                          3.59912    0.76208 803.51664
## bearded                                      5.73465    0.55685 915.00673
## Pregancy_Ambition_item_removed:masc          0.07889    0.12827 915.00098
## Pregancy_Ambition_item_removed:age          -1.43721    0.78290 446.04012
## masc:age                                     0.38552    0.13419 915.00098
## Pregancy_Ambition_item_removed:bearded      -0.79641    0.55578 915.00674
## age:bearded                                 -1.17792    0.58146 915.00674
## Pregancy_Ambition_item_removed:masc:age     -0.21222    0.13580 915.00098
## Pregancy_Ambition_item_removed:age:bearded   0.21148    0.58841 915.00675
##                                            t value Pr(>|t|)
## (Intercept)                                 38.324 9.38e-13 ***
## Pregancy_Ambition_item_removed               2.272  0.02364 *
## masc                                        10.962  < 2e-16 ***
## age                                          4.723 2.75e-06 ***
## bearded                                     10.298  < 2e-16 ***
## Pregancy_Ambition_item_removed:masc          0.615  0.53870
## Pregancy_Ambition_item_removed:age          -1.836  0.06706 .
## masc:age                                     2.873  0.00416 **
## Pregancy_Ambition_item_removed:bearded      -1.433  0.15221
## age:bearded                                 -2.026  0.04308 *
## Pregancy_Ambition_item_removed:masc:age     -1.563  0.11846
## Pregancy_Ambition_item_removed:age:bearded   0.359  0.71937
## ---
## Signif. codes:  0 '***' 0.001 '**' 0.01 '*' 0.05 '.' 0.1 ' ' 1
##
## Correlation of Fixed Effects:
##                      (Intr) Pr_A__ masc   age    beardd
## Prgncy_Am__           0.179
## masc                 -0.009  0.000
## age                   0.160  0.385 -0.001
## bearded               0.040  0.002  0.095  0.006
## Prgncy_Ambtn_tm_rmvd:m   0.000 -0.011  0.041 -0.004  0.004
## Prgncy_Ambtn_tm_rmvd:g   0.372  0.136 -0.004  0.320  0.020
## masc:age             -0.001 -0.004  0.118 -0.012  0.011
## Prgncy_Ambtn_tm_rmvd:b   0.002  0.051  0.004  0.019  0.041
## age:bearded           0.005  0.019  0.011  0.052  0.118
## Prgncy_Ambtn_tm_rmvd:m: -0.003 -0.001  0.378 -0.004  0.036
## Prgncy_Ambtn_tm_rmvd:g:  0.015  0.006  0.036  0.016  0.378
##                      Prgncy_Ambtn_tm_rmvd:m Prgncy_Ambtn_tm_rmvd:g
## Prgncy_Am__
## masc
## age
## bearded
## Prgncy_Ambtn_tm_rmvd:m
## Prgncy_Ambtn_tm_rmvd:g  -0.001
## masc:age              0.372                  -0.004
```

```
## Prgncy_Ambtn_tm_rmvd:b   0.095                 0.006
## age:bearded              0.035                 0.016
## Prgncy_Ambtn_tm_rmvd:m:  0.109                -0.011
## Prgncy_Ambtn_tm_rmvd:g:  0.010                 0.052
##                         masc:g Prgncy_Ambtn_tm_rmvd:b ag:brd
## Prgncy_Am__
## masc
## age
## bearded
## Prgncy_Ambtn_tm_rmvd:m
## Prgncy_Ambtn_tm_rmvd:g
## masc:age
## Prgncy_Ambtn_tm_rmvd:b   0.035
## age:bearded              0.095  0.372
## Prgncy_Ambtn_tm_rmvd:m:  0.312  0.010                  0.030
## Prgncy_Ambtn_tm_rmvd:g:  0.030  0.109                  0.312
##                         Prgncy_Ambtn_tm_rmvd:m:
## Prgncy_Am__
## masc
## age
## bearded
## Prgncy_Ambtn_tm_rmvd:m
## Prgncy_Ambtn_tm_rmvd:g
## masc:age
## Prgncy_Ambtn_tm_rmvd:b
## age:bearded
## Prgncy_Ambtn_tm_rmvd:m:
## Prgncy_Ambtn_tm_rmvd:g:  0.095
## convergence code: 0
## singular fit
```

Father/Partner Beardedness on Preferences

```
model4 <- lmer(rating ~     fathers_beards*masc*sl_term + fathers_beards*bearded*sl_term +
                        partners_beards*masc*sl_term + partners_beards*bearded*sl_term +
                        (1 + masc*sl_term + bearded*sl_term | ID) +
                        (1 + partners_beards + fathers_beards | stimuli),
            data = data.analysis)

save(model4,file = "model4.Rdata")
```

```
summary(model4)
```

```
## Linear mixed model fit by REML. t-tests use Satterthwaite's method [
## lmerModLmerTest]
## Formula:
## rating ~ fathers_beards * masc * sl_term + fathers_beards * bearded *
##     sl_term + partners_beards * masc * sl_term + partners_beards *
##     bearded * sl_term + (1 + masc * sl_term + bearded * sl_term |
##     ID) + (1 + partners_beards + fathers_beards | stimuli)
##    Data: data.analysis
##
## REML criterion at convergence: 271185.2
```

```
##
## Scaled residuals:
##     Min      1Q  Median      3Q     Max
## -5.2348 -0.4866 -0.0461  0.4398  5.7796
##
## Random effects:
##  Groups   Name            Variance  Std.Dev. Corr
##  ID       (Intercept)     408.11772 20.2019
##           masc              8.56826  2.9272   0.04
##           sl_term          55.63157  7.4587   0.07 -0.03
##           bearded         212.97484 14.5937   0.04  0.06  0.02
##           masc:sl_term      0.37885  0.6155   0.56  0.74  0.38  0.25
##           sl_term:bearded  96.58490  9.8278   0.02  0.04  0.15  0.25  0.16
##  stimuli  (Intercept)       1.94879  1.3960
##           partners_beards   0.02754  0.1659   0.99
##           fathers_beards    0.14686  0.3832  -0.99 -0.96
##  Residual                 178.10240 13.3455
## Number of obs: 33000, groups:  ID, 550; stimuli, 3
##
## Fixed effects:
##                                  Estimate Std. Error        df t value
## (Intercept)                       36.2837     1.1827    9.0637  30.679
## fathers_beards                    -0.2152     0.8889  252.3013  -0.242
## masc                               1.5637     0.1496  547.3467  10.454
## sl_term                            0.6018     0.3507  546.9887   1.716
## bearded                            5.1459     0.6401  546.9911   8.039
## partners_beards                   -0.1168     0.8643  513.3250  -0.135
## fathers_beards:masc               -0.2846     0.1488  547.3467  -1.913
## fathers_beards:sl_term             0.6394     0.3489  546.9887   1.833
## masc:sl_term                       0.6277     0.1665 7715.8993   3.769
## fathers_beards:bearded             0.2140     0.6367  546.9911   0.336
## sl_term:bearded                    0.8758     0.5124  547.0085   1.709
## masc:partners_beards               0.2467     0.1484  547.3467   1.661
## sl_term:partners_beards            0.6251     0.3481  546.9887   1.796
## bearded:partners_beards            5.6702     0.6353  546.9911   8.926
## fathers_beards:masc:sl_term       -0.1104     0.1657 7715.8992  -0.666
## fathers_beards:sl_term:bearded     0.8233     0.5097  547.0085   1.615
## masc:sl_term:partners_beards      -0.0646     0.1653 7715.8993  -0.391
## sl_term:bearded:partners_beards    1.3546     0.5085  547.0085   2.664
##                                 Pr(>|t|)
## (Intercept)                     1.80e-10 ***
## fathers_beards                  0.808953
## masc                             < 2e-16 ***
## sl_term                         0.086763 .
## bearded                         5.62e-15 ***
## partners_beards                 0.892567
## fathers_beards:masc             0.056308 .
## fathers_beards:sl_term          0.067392 .
## masc:sl_term                    0.000165 ***
## fathers_beards:bearded          0.736938
## sl_term:bearded                 0.087976 .
## masc:partners_beards            0.097187 .
## sl_term:partners_beards         0.073059 .
## bearded:partners_beards          < 2e-16 ***
```

```
## fathers_beards:masc:sl_term     0.505224
## fathers_beards:sl_term:bearded  0.106856
## masc:sl_term:partners_beards    0.695944
## sl_term:bearded:partners_beards 0.007954 **
## ---
## Signif. codes:  0 '***' 0.001 '**' 0.01 '*' 0.05 '.' 0.1 ' ' 1
```

```
##
## Correlation matrix not shown by default, as p = 18 > 12.
## Use print(x, correlation=TRUE)  or
##     vcov(x)        if you need it
```

```
## convergence code: 1
## singular fit
```

Plot Data

```
plot.data <- data.analysis %>%
  gather(key = "scale",value = "disgust",Tybur_Pathogen,Tybur_Sexual,Tybur_Moral,Heebie_Jeebies) %>%
  mutate(scale = recode(scale,"Tybur_Pathogen" = "Pathogen Disgust",
                      "Tybur_Sexual" = "Sexual Disgust",
                      "Tybur_Moral" = "Moral Disgust",
                      "Heebie_Jeebies" = "Ectoparasite Avoidance"),
         bearded = recode(bearded, "-0.5" = "Clean-Shaven",
                          "0.5" = "Bearded"),
         sl_term = recode(sl_term,"-0.5" = "Short-Term Attractiveness",
                          "0.5" = "Long-Term Attractiveness"),
         masc = recode(round(masc,1), `-1.3` = "40%",
                                      `-.6` = "80%",
                                      `0` = "100%",
                                      `.6` = "130%",
                                      `1.3` = "160%"),
         masc = factor(masc,levels = c("40%","80%","100%","130%","160%")),
         rs = recode(rs,"-0.5" = "Single/Dating",
                      "0.5" = "Married/Committed Relationship"))

ggplot(data = plot.data,aes(x = disgust,y = rating,group = as.factor(bearded),colour = as.factor(bearde
  geom_smooth(method = "lm") +
  facet_wrap(~scale) +
  xlab("Disgust Scale") +
  ylab("Attractiveness Rating") +
  labs(colour = "Facial Hair")
```

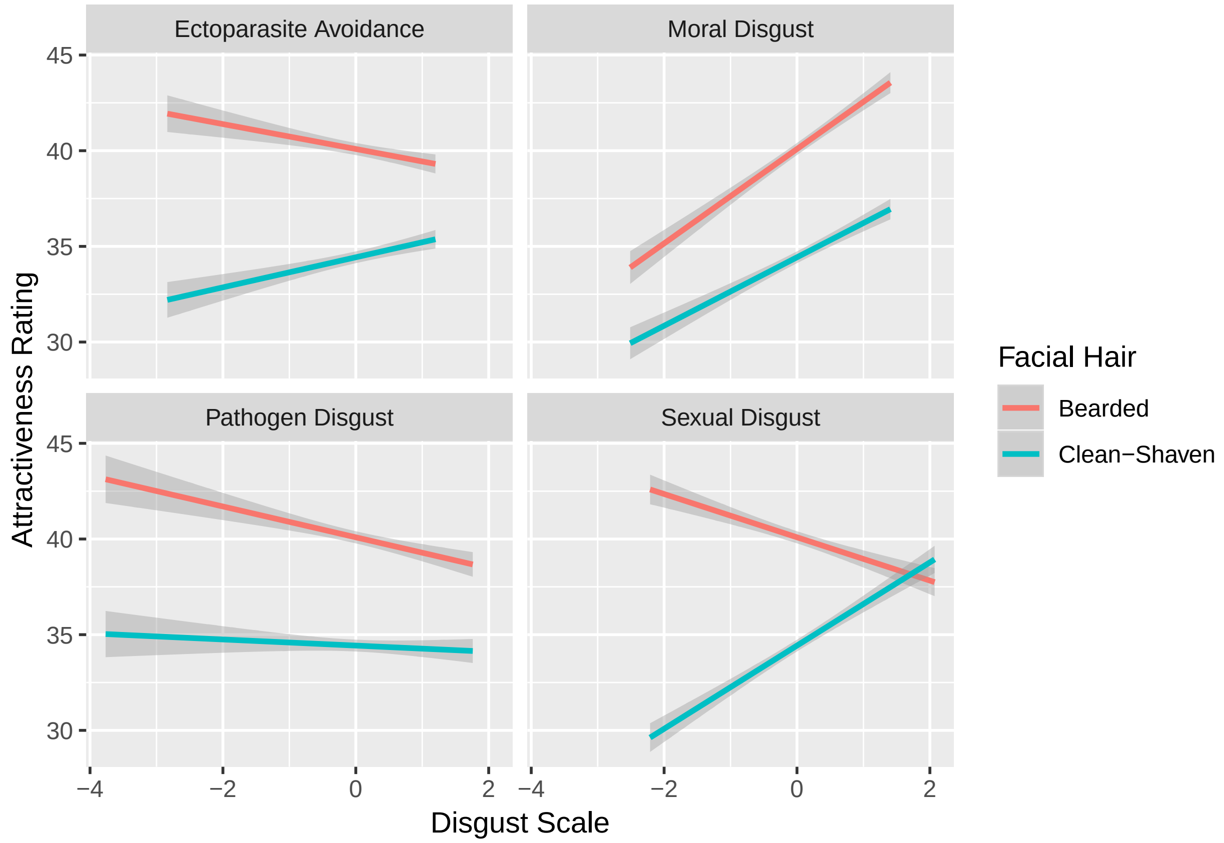


```
ggsave("bearded.jpg")
```

```
## Saving 6.5 x 4.5 in image
```

```
ggplot(data = plot.data,aes(x = disgust,y = rating,group = masc,colour = masc)) +
  geom_smooth(method = "lm") +
  scale_colour_manual(values = c("red","orange","yellow","green","blue")) +
  facet_wrap(~scale) +
  xlab("Scale") +
  ylab("Attractiveness Rating") +
  labs(colour = "Facial Masculinity")
```

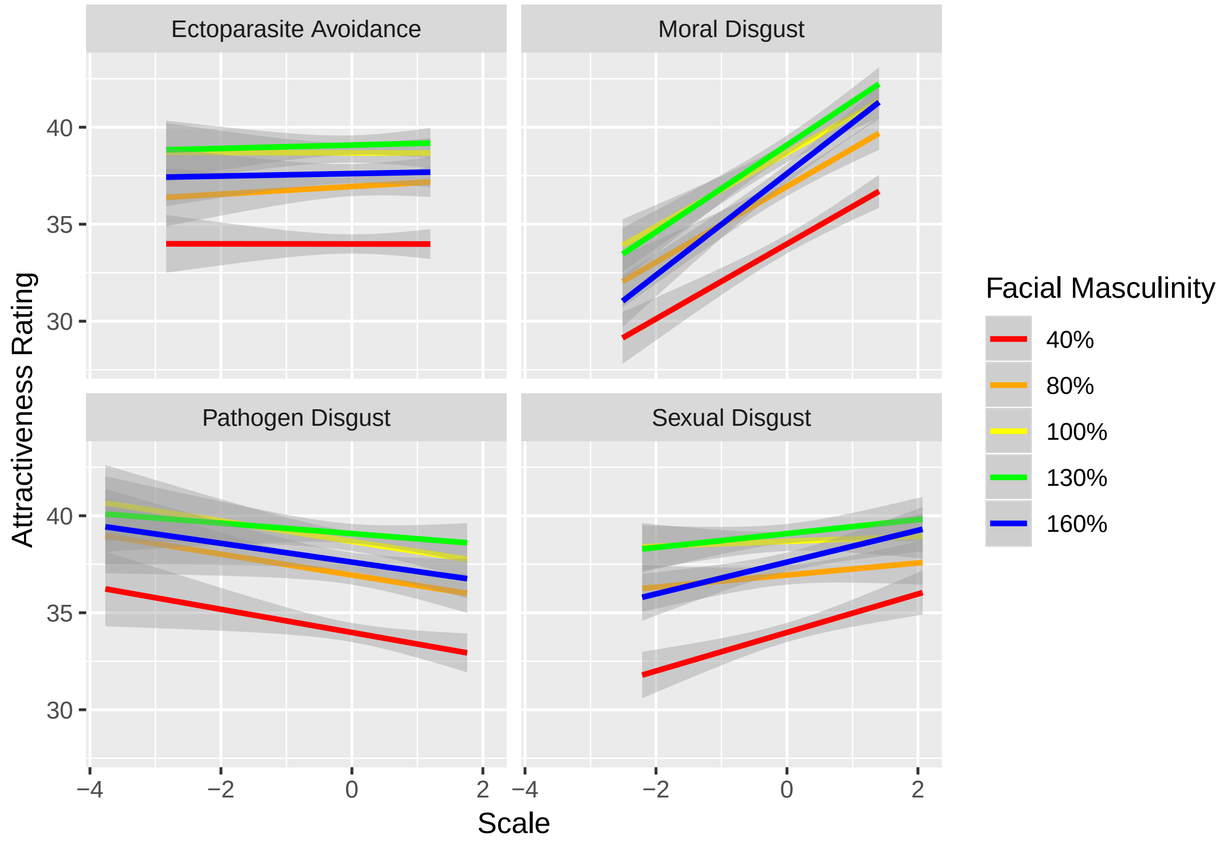


```r
ggsave("masc.jpg")
```

```
## Saving 6.5 x 4.5 in image
```

```r
ggplot(data = plot.data,aes(x = disgust,y = rating,group = as.factor(sl_term),colour = as.factor(sl_term
  geom_smooth(method = "lm") +
  facet_wrap(~scale) +
  xlab("Scale") +
  ylab("Attractiveness Rating") +
  labs(colour = "Relationship Context")
```

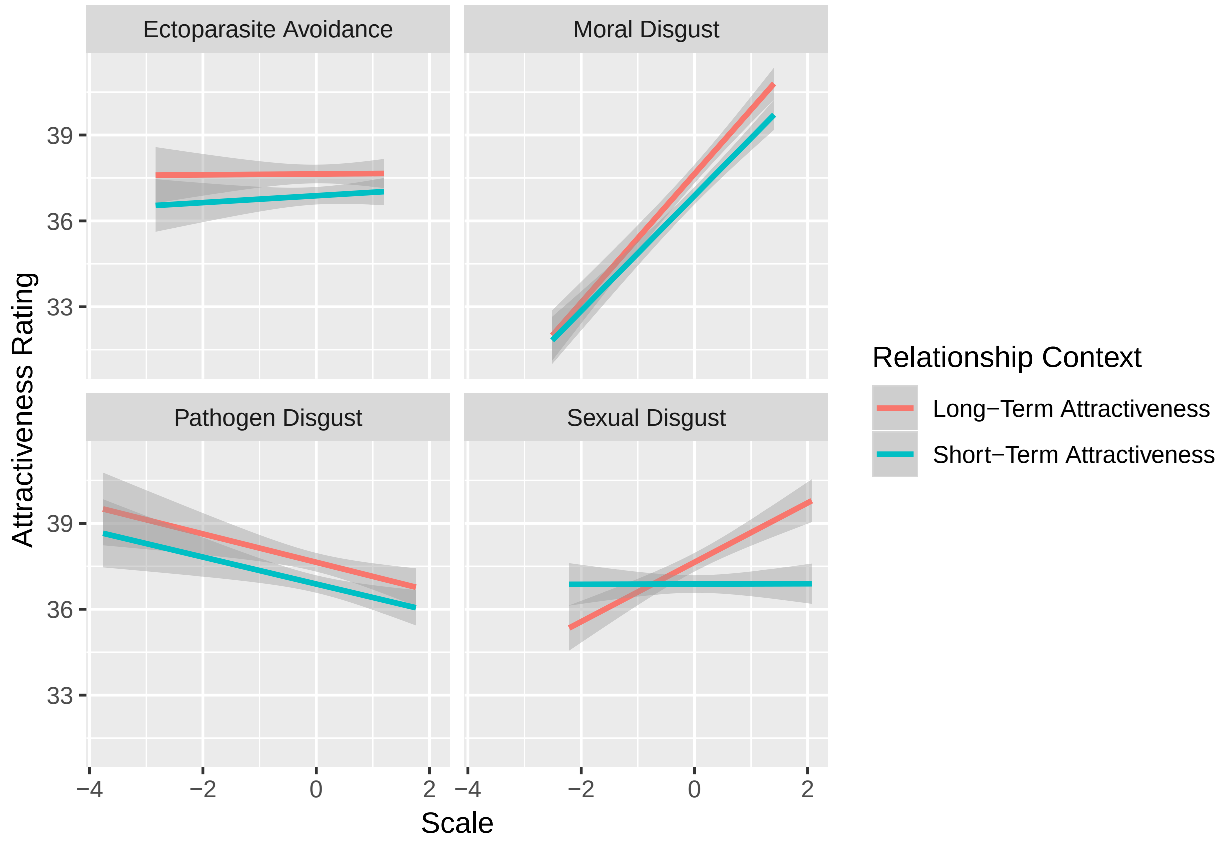


```
ggsave("sl_term.jpg")
```

```
## Saving 6.5 x 4.5 in image
```

```
ggplot(data = plot.data,aes(x = Pregancy_Ambition_item_removed,y = rating,group = as.factor(rs),colour =
  geom_smooth(method = "lm") +
  facet_wrap(~ bearded) +
  xlab("Pregnancy Ambition") +
  ylab("Attractiveness Rating") +
  labs(colour = "Relationship Status")
```

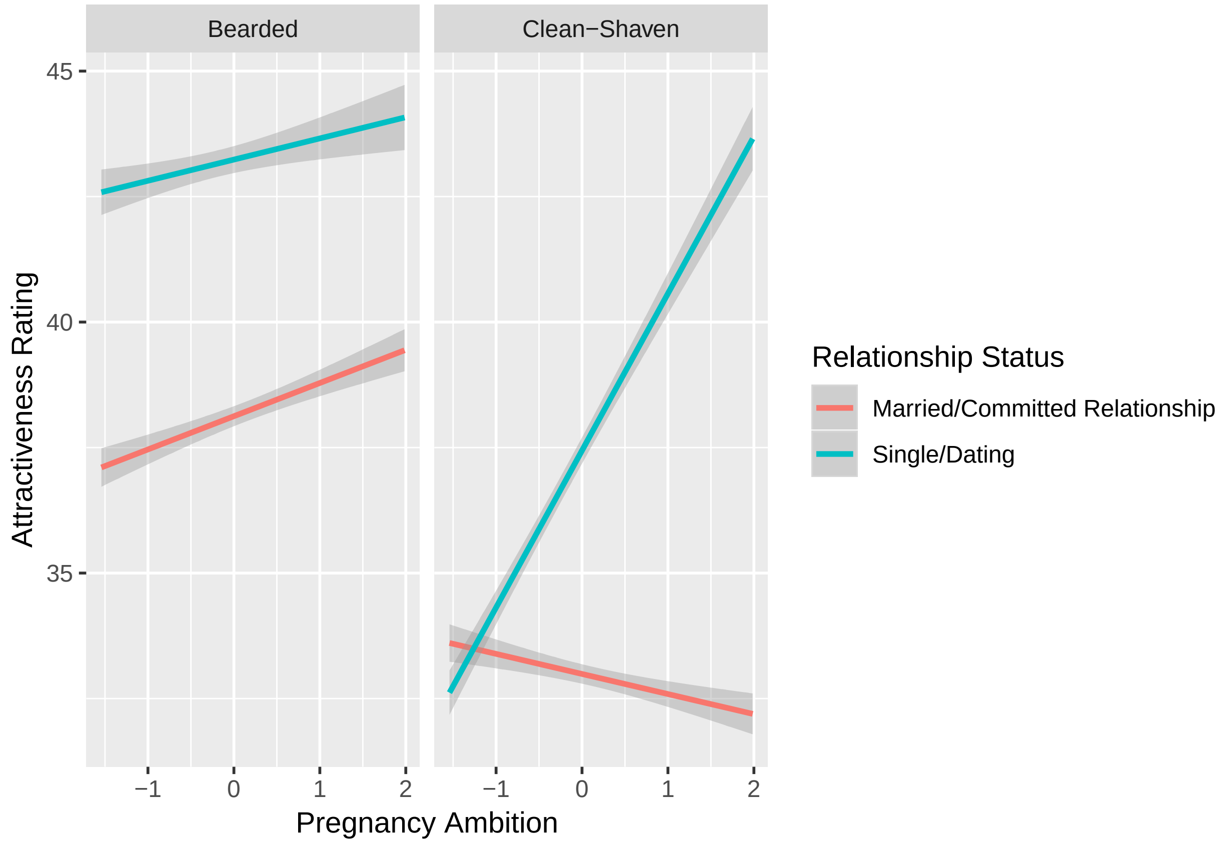


```r
ggsave("pregam_rs.jpg")
```

```
## Saving 6.5 x 4.5 in image
```
